# Anti-sickling Activity of Ursolic Acid Isolated from the Leaves of *Ocimum gratissimum* L. (Lamiaceae)

**DOI:** 10.1007/s13659-015-0070-6

**Published:** 2015-09-09

**Authors:** Dorothée Dinangayi Tshilanda, Damase NguwoVele Onyamboko, Philippe Babady-Bila, Koto-te-Nyiwa Ngbolua, Damien ShaTshibey Tshibangu, Eddy dia Fita Dibwe, Pius Tshimankinda Mpiana

**Affiliations:** Faculté des Sciences, Université de Kinshasa, B.P. 190, Kinshasa XI, Democratic Republic of the Congo; Institute of Natural Medicine, University of Toyama, 2630-Sugitani, Toyama, 930-0194 Japan; Bila Nutraceuticals Company, 650 Portland Street Market, PO Box 26056, Dartmouth, NS B2W 6P3 Canada

**Keywords:** *Ocimum gratissimum*, Sickle cell disease, Antisickling activity, Ursolic acid

## Abstract

**Abstract:**

The present study reports in vitro anti-sickling activity and phytochemical analyses of the leaves of *Ocimum gratissimum*. Biological testing revealed that the plant extracts possess antisickling effects. The combination of spectroscopic techniques: 1D-NMR, 2D-NMR and MS revealed that ursolic acid is the major biologically active compound of *O.* gratissimum (Silva et al. in Molecules 13:2482–2487, 2008; Kedar et al. J Food Drug Anal 20:865–871, 2012). This study is the first report of the antisickling activity of ursolic acid isolated from *O. gratissimum*. The pharmaceutical relevance of findings from this study derives from the possibility of integrating *O. gratissimum* as an antisickling plant in the pharmacopoeia of Democratic Republic of the Congo. The identification of the active principle could enhance the standardization of antisickling recipe.

**Graphical Abstract:**

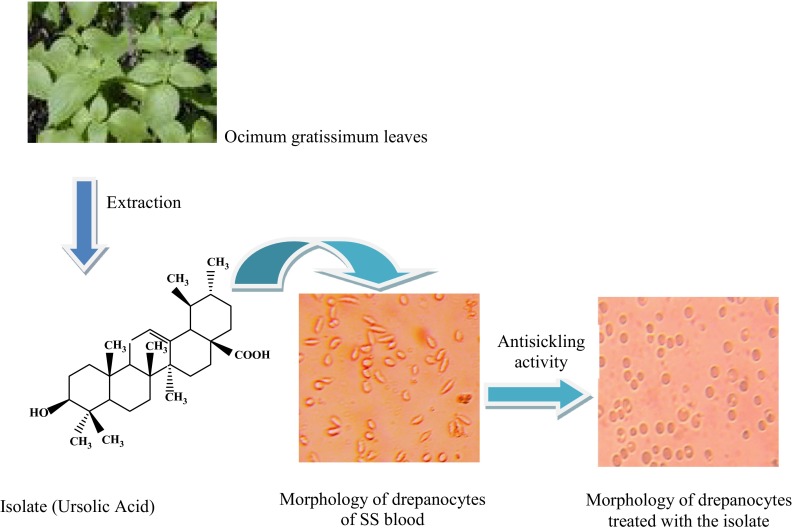

## Introduction

Sickle cell disease (SCD) or sickle cell anemia is a blood disease that affects people originated from tropical areas particularly the black people [3, 4]. The disease is due to a replacement of glutamic acid located in the sixth position of the β chain of hemoglobin by valine [5, 6]. The substitution of this amino acid alters both the affinity of hemoglobin for oxygen and its solubility in hypoxic conditions [7–9]. The decrease of solubility causes polymerization of hemoglobin and the sickling of red blood cells and constitutes the pathophysiological basis of the symptoms of SCD [5, 10]. Each year about five million people are affected by SCD in the world. In some parts of Africa, the rate of sickle cell trait reaches 20 % of the population with a high prevalence in Central Africa (from 25 to 30 %) [5].

More than 200000 sicklers are born each year in Africa [11]. Two percent of the Congolese populations are affected by this disease [12, 13]. The majority of children affected by SCD die before the age of 5 years if they do not received medical care [4, 5, 7, 14]. The percentage of people suffering from this disease continues to grow making SCD a real public health problem in endemic regions [11]. This constitutes a great challenge for the search of an affordable treatment.

Several managing SCD therapy strategies have been proposed including the bone marrow transplantation, gene therapy, repeated blood transfusions and treatment with hydroxyurea. However, some of these treatments are ineffective or very expensive for the less fortunate African populations, or may constitute a risk of HIV/AIDS infections [4, 15, 16]. Recently, several studies have reported the use of medicinal plants for the treatment of SCD [17–20]. In DR Congo, Mpiana and coworkers reported that more than 70 medicinal plant species used in folk medicine for the treatment of SCD display in vitro anti-sickling activity [6, 12, 13, 15, 21–24]. The bioactivity of these plants is usually due to the presence of anthocyanins, organic acids or butyl stearate [2, 4, 6, 13, 21–26].

In a previous investigation we reported the isolation of an anti-sickling molecule (butyl stearate) from *Ocimum basilicum* [27]. The present work aims to assess the antisickling activity of the leaves of another species of Ocimum genus: *O. gratissimum* and to isolate and elucidate the structure of the eventual bioactive compounds.

## Results and Discussion

### Phytochemical Screening and Extraction Yield

The result of the phytochemical screening performed on the leaves extracts revealed the presence of polyphenols (flavonoids, anthocyanins, leucoanthocyanins, tannins, quinones), alkaloids, saponins, triterpenoids and steroids. The ethyl acetate extracts from *O. gratissimum* showed a significant antisickling activity.

### Anti-sickling Activity of Ethyl Acetate Crude Extracts of *O. gratissimum* Leaves

**Fig. 1:**
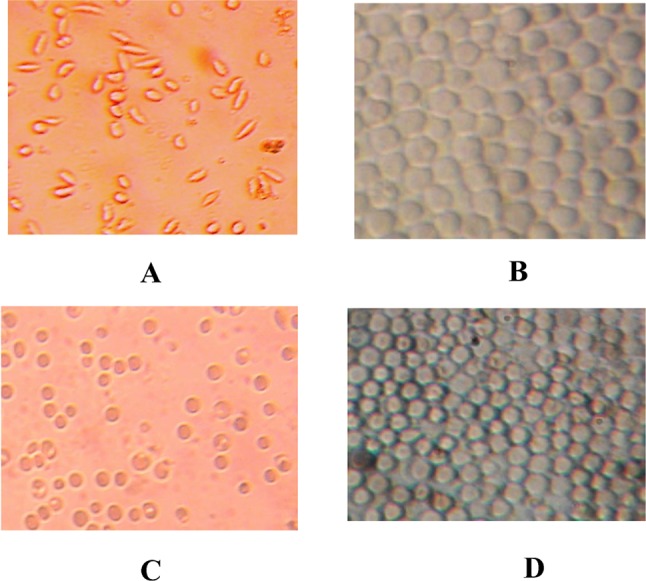
Morphology of drepanocytes of SS blood before (**a** negative control) and after treatment with betulinic acid (**b** positive control: 20 µg/mL), ethyl acetate extract (**c** 50 µg/mL), and isolate (**d** 20 µg/mL) [NaCl 0.9%; Na_2_S_2_O_5_ 2%, ×500]

The Fig. 1 gives the sickle erythrocytes phenotype of SS blood alone (negative control: **A**), SS blood treated with betulinic acid (positive control: **B**) and SS blood treated with ethyl acetate extract (**C**) or the isolate (**D**) respectively.

The bioactivity was assessed based on the sickling of red blood cells (RBCs) as phenotype marker (Fig. 1a). This figure shows that the majority of RBCs are elongated (sickled). This confirms that the used blood is SS one. When betulinic acid (positive control) is added, RBCs show circular (biconcave) and normal shape (Fig. 1b). Ethyl acetate extract and the isolate displayed remarkable sickling inhibitory effects. Treated sickle erythrocytes displayed a very similar phenotype/morphology than that of the normal erythrocytes (Fig. 1c) and (Fig. 1d). The morphological change is similar to that of the positive control, indicating the antisickling effect of *O. gratissimum*. Thus, this plant could serve according to chemo-taxonomical approach as promising source of anti-sickling new lead compounds.

The calculated average of radius, perimeter and surface of drepanocytes before and after treatment with the isolated compound are given in Table 1.

**Table 1:** Average values of radius, perimeter and surface of erythrocytes before and after treatment the isolated compound (ICD) of *O. gratissimum*

Measured parameters	Untreated SS RBC_s_	SS RBC_s_ (+ICD)
Radius (µm)	–	3.1 ± 0.3
Perimeter (µm)	31.7 ± 1.5	18.3 ± 1.1
Surface (µm^2)^	19.2 ± 1.0	31.8 ± 1.7

*RBC*_*S*_ red blood cells

It is deduced from this table that the normalization (antisickling activity) causes the reappearance of the circular form of the erythrocytes, the diminution of their perimeter and the increase in cellular surface. These results confirm those already reported by our research team for anthocyanins and organic acids such as maslinic and lunilaric acids from plants used in traditional medicine for the management of sickle cell anemia [3, 28].

Sickle cell disease is a blood disease characterized by the aggregation of hemoglobin S under hypoxic conditions. Its symptoms are erythrocytes shape modification and anemia. The need to search for new drugs with low toxicity is the new challenges. The plant kingdom could serve as source of the antisickling new lead compounds as herein demonstrated. Recent findings indicate that plant polar extracts are more active on sickle blood cells that non polar one [4, 17, 21–24].

Since ethyl acetate extract displayed significant anti-sickling activity, it was subjected to chromatographic bio-guided fractionation to afford an active isolate.

The EI-MS spectrum of the isolate showed molecular ion peak at *m*/*z* 456 that corresponds to molecular formula C_30_H_48_O_3_, with other peaks at *m*/*z* = 248 (base peak), 203, and 189 characteristics of a Retro-Diels–Alder typical fragmentation of ring C ursolic and oleanolic acids. Other peaks were observed at *m*/*z*: 438 (7.30), 392 (38.60), 207 (39.17), 203 (72.10), 233 (6.08), 189 (47.24) and 119 (30.71), with one small impurity peak at *m*/*z* = 662.

The ^1^H NMR date of the isolate showed the presence of five sharp singlet methyl groups (0.78, 0.81, 0.92. 0.98 and 1.08 ppm) and two doublet methyl groups (0.86 and 0.94 ppm) in the region of higher field, characteristic of ursane-type triterpenes skeleton [29]. A weak singlet peak at *δ*_H_ 1.14 ppm and a broad singlet peak at 1.26 ppm were also observed. The peak at *δ*_H_ 1.14 ppm is generally characteristic for methyl group-27 of oleanane triterpene skeleton and the broad weak peak at *δ*_H_ 1.26 ppm suggests the presence of a long chain methylene protons –(CH_2_)n– in the isolate. In the region of lower field, the peak at *δ*_H_ 3.43 ppm represents H-3 due to the attachment of a hydroxyl group to C-3 and an olefinic proton peak at *δ*_H_ 5.29 ppm was assigned to H-12 of a triterpene.

 The ^13^C-NMR and DEPT ^13^C-NMR data (Table 2) indicate the presence of double bond Δ12 at *δ*_C_ 125.5 ppm (=CH) and 138.1 ppm (–C=), suggesting that the major compound belongs to the ursane-type triterpene group [29]. The ^13^C-NMR spectrum indicates the presence of the following peaks characteristic of the triterpene groups *α*- and *β*-amyrine: *δ*_C_ = 79.1, 122.3, 125.5, 138.1 and 181.0 ppm. The peak at *δ* 79.1 ppm can be assigned at a C-3 (CH–O–) of a triterpenoid; the peaks 125.5 and 138.1 ppm are characteristics of the double bond Δ12 of triterpenoids of ursane type (*α*-amyrin) [29]. The weak peak at *δ*_C_ 122.3 ppm is characteristic of C-12 of double bond Δ12 for oleanane-type triterpenoids (*β*-amyrine) although the characteristic peak of C-13 of these double bonds is not observed. The peak at *δ*_C_ 181 ppm corresponds to a free carboxylic group (–COOH).

**Table 2:** ^1^H and ^13^C NMR data

C	^1^H (*δ*)	Multiplicity	^13^C (*δ*)	Dept
1			38.7	CH_2_
2			29.7	CH_2_
3	3.43	(1H, *dd*, *J* = 10.0 Hz, 4.5 Hz)	79.1	CH
4	–		39.0	C
5	0.72	(1H, *m*)	55.1	CH_2_
6			18.4	CH_2_
7			33.1	CH_2_
8	–		39.4	C
9			47.5	CH
10	–		36.7	C
11			23.3	CH_2_
12	5.29	(1H, *t,* *J* = 3.6 Hz)	125.5	CH=
13	–		138.1	C=
14			41.3	C
15			28.1	CH_2_
16			24.3	CH_2_
17	–		47.8	C
18	2.20	(1H, *d*, *J* = 11.7 Hz,)	52.7	CH
19			39.2	CH
20			38.9	CH
21			30.7	CH_2_
22			36.9	CH_2_
23	0.98	3H (s)	28.1	CH_3_
24	0.78	3H (s)	15.7	CH_3_
25	0.81	3H (s)	15.5	CH_3_
26	0.92	3H (s)	17.0	CH_3_
27	1.08	3H (s)	23.6	CH_3_
28	–	–	181.0	O-C=O
29	0.86	3H, *d* : J = 6.5 Hz	17.1	CH_3_
30	0.94	3H, *d* : J = 6.5 Hz	21.2	CH_3_

In the COSY spectrum H-18 (*δ*_H_ 2.20 ppm) showed cross peaks with H-19 (*δ*_H_ 1.38 ppm) and with H-20 (*δ*_H_ 1.50 ppm) while H-20 was coupled to H-19. The COSY spectrum revealed also that H-2 was coupled to H-3 and H-12 was coupled to H-11. HMQC data indicated correlations by the cross peak between the following H and CH-3 (3.43 ppm) and C-3 (79.1 ppm); H-5 (0.72 ppm) and C-5 (55.3 ppm); H-6 (1.58 ppm) and C-6 (18.4 ppm); H-9 (1.50 ppm) and C-9 (47.6 ppm); H-12 (5.29 ppm) and C-12 (125.5 ppm); H-18 (2.20 ppm) and C-18 (52.7 ppm). The others HMQC correlations are displayed in Table 2.

In HMBC spectrum the presence of a trisubstituted olefin between C-12 and C-13 was confirmed by the correlations observed for H-11/C-12, H-11/C-13, H-18/C-13 and H-27/C-13. HMBC spectrum showed also correlations by the cross peak between methyl proton peaks at *δ* 0.98 ppm (H-23) and 0.78 ppm (H-24) with carbon signal at *δ* 39.0 ppm (C-4). The methyl group at *δ* 0.86 ppm (H-29) correlated to carbons at *δ* 52.7 ppm (C-18) and *δ* 39.2 ppm (C-20). The methyl group at *δ* 0.94 ppm (H-30) correlated to the signal of the carbons at *δ* 39.2 ppm (C-19) and 38 ppm (C-20). The complete assignments of NMR data are displayed in Tables 2.

Thus, these spectroscopic data indicate that the major compound in our isolate is ursolic acid contaminated by trace of an oleanane-type triterpene and trace of an unidentified fatty acid ester.

Ursolic acid is a well-known triterpene found in many plants and herbs [30–37]. It exhibits a wide range of biological activities ant-inflammatory, hepatoprotective, analgesic, antimicrobial, antimycotic, virostatic, anti-diabetic, anti-malarial, anti-oxidative. immunomodulatory, diuretic, anti- spasmodic, anti-atherosclerotic, anti-tumor, anti HIV, activity anti-*Mycobacterium tuberculosis* and anti-leishmanial activities [34, 37, 38].

The present study reports of the first time the antisickling activity of the ursolic acid isolated from *O. gratissimum* leaves.

## Conclusion

The present research work depicts *in vitro* anti-sickling activity and phytochemical analyses of the leaves of *O. gratissimum*. Biological testing revealed that the ethyl acetate extracts and its isolate possess anti-sickling effects. The combination of spectroscopic techniques: 1D-NMR (^1^H-NMR, ^13^C-NMR), 2D-NMR (COSY, HMBC, HMQC) and MS revealed that ursolic acid is the major biologically active compound of *O.* gratissimum [1, 2]. This is the first report of the anti-sickling activity of ursolic acid isolated from *O. gratissimum*.

The pharmaceutical relevance of findings from this study open the possibility of integrating *O. gratissimum* as an anti-sickling plant in the pharmacopoeia of Democratic Republic of the Congo. The identification of the active principle could enhance the standardization of anti-sickling recipe.

## Experimental Section

### General Experimental Procedures

1D-NMR (^1^H-NMR, ^13^C-NMR) and 2D-NMR (COSY, HMQC, HMBC) data were measured on a JEOL JNM-LA400 spectrometer with TMS used as internal Standard (400 MHz for ^1^H-NMR and 100 MHz for ^13^C-NMR). The chemical shifts values (*δ*) are expressed in ppm units, and coupling constants (*J*) are reported in Hz. All spectra were taken at room temperature using deuterated chloroform (CDCl_3_) as solvent. LREIMS data were made using a JEOL JMS-700T mass spectrometer.

Thin layer chromatography (TLC) was performed on precoated silica gel 60 TLC plates (DC-Fertigplatten Kieselgel 60 F_254_ and DC-Alufolien Kieselgel 60 F_254_). Silica gel for column chromatography (Kiesel gel brand 60 F_254_ 0.2–0.5/35–70 mesh ASTM) was used for column chromatography. A rotary evaporator (Büchi Re 120 model), an oven (Brand MEMMERT model), and a UV lamp (type CAMAG) have also been used.

### Plant Material

*O. gratissimum* leaves were harvested in the University of Kinshasa surroundings from May to June 2009 and dried at room temperature for fourteen days prior to extraction. The plant material was authenticated by Mr. Nlandu. A voucher specimen (number 8016) is deposited in the herbarium of the “Institut National des Recherches Agronomiques (INERA)”, Faculty of Sciences, University of Kinshasa.

### Biological Material

The homozygous hemoglobin S blood sample used to evaluate the antisickling activity was obtained from patients attending the “Centre de Médecine Mixte et d’Anémie SS”, located in Kinshasa area, DR Congo. None of the patients had been transfused recently with homozygous hemoglobin A blood. All antisickling bioassays were carried out with freshly collected blood. In order to confirm their SS nature, the above-mentioned blood samples were first characterized by hemoglobin electrophoresis on cellulose acetate gel at pH 8.5 as previously reported [8]. They were found to be SS blood and were then stored in a refrigerator at 4 °C.

### Anti-sickling Activity

The blood sample was mixed with the crude extract or the isolate, using physiological saline as solvent. The Emmel test was performed to evaluate the antisickling activity as previously reported [4]. Microscopic images were examined under an optical microscope brand Bresser Biolux NV 20X-1280X Model and images were processed using MOTIC Images 2000 version 1.3 software.

The experiments were carried out under hypoxic conditions (P_O2_ < 45 mmHg) and the treatment lasts as long as the slides are well kept. After 2 h under these conditions, the blood samples were observed under an optical microscope and the number of observed erythrocytes was determined. We consider that an extract or compound has a high activity if at least 70 % of erythrocytes are normalized [12]. In this study, the percentages of RBCs that showed circular and normal shape were 95% (positive control), 94% (isolate), and 90% (ethyl acetate).

The anti-sickling activity determined in this study is dose dependent as we used the same experimental procedure than in our previously published works [39, 40].

### Bio-guided Extraction

The dried and powdered plant material (leaves, 1000 g) was successively soaked twice during 48 h in *n*-hexane, dichloromethane, ethyl acetate and methanol at room temperature (3 L x 2 each). After filtration, each extract was concentrated to dryness under reduced pressure using a rotary evaporator and dried at 50 °C in the oven, to afford *n*-hexane (17.63 g: 1.76 %), dichloromethane (26.66 g: 2.67 %), ethyl acetate (12.56 g: 1.26 %) and methanol (33.10 g: 3.31 %) extracts. Chemical screening was performed on aqueous suspensions and organic solutions for each extract, using a well-known protocol [12, 27]. Ethyl acetate and methanol extracts exhibited an antisicking activity, with ethyl acetate extract showing a stronger activity.

### Fractionation of the Ethyl Acetate Extract

The ethyl acetate was submitted to a TLC analysis on silica gel plates with the mixture of *n*-hexane-ethyl acetate (6:4, v/v) as eluting system. Twelve spots (including one major spot) were detected using a UV lamp (at 254 and 366 nm) and spraying sulfuric acid 20 % in ethanol followed by heating [41, 42]. It was then fractionated on a silica gel column eluting with the same system used for TLC analysis (*n*-hexane-ethyl acetate 6:4, v/v) to give 4 fractions: F1, F2, F3 and F4. The column was further washed with methanol to afford a pale yellow powder product (fraction F5) after evaporation of methanol. This product exhibited the strongest antisickling activity. Its TLC analysis using dichloromethane as eluent displayed a large spot (R_f_: 0.5).
